# A Fiber-Optic Sensor for Acoustic Emission Detection in a High Voltage Cable System

**DOI:** 10.3390/s16122026

**Published:** 2016-11-30

**Authors:** Tongzhi Zhang, Fufei Pang, Huanhuan Liu, Jiajing Cheng, Longbao Lv, Xiaobei Zhang, Na Chen, Tingyun Wang

**Affiliations:** Key Laboratory of Specialty Fiber Optics and Optical Access Networks, Shanghai University, 149 Yanchang Road, Shanghai 200072, China; clove@shu.edu.cn (T.Z.); hhliu@shu.edu.cn (H.L.); jiajingcheng@i.shu.edu.cn (J.C.); lvlongbao@i.shu.edu.cn (L.L.); xbzhang@shu.edu.cn (X.Z.); na.chen@shu.edu.cn (N.C.); tywang@shu.edu.cn (T.W.)

**Keywords:** fiber-optic sensors, interferometry, acoustic emission, partial discharge, GIS

## Abstract

We have proposed and demonstrated a Michelson interferometer-based fiber sensor for detecting acoustic emission generated from the partial discharge (PD) of the accessories of a high-voltage cable system. The developed sensor head is integrated with a compact and relatively high sensitivity cylindrical elastomer. Such a sensor has a broadband frequency response and a relatively high sensitivity in a harsh environment under a high-voltage electric field. The design and fabrication of the sensor head integrated with the cylindrical elastomer is described, and a series of experiments was conducted to evaluate the sensing performance. The experimental results demonstrate that the sensitivity of our developed sensor for acoustic detection of partial discharges is 1.7 rad/(m⋅Pa). A high frequency response up to 150 kHz is achieved. Moreover, the relatively high sensitivity for the detection of PD is verified in both the laboratory environment and gas insulated switchgear. The obtained results show the great potential application of a Michelson interferometer-based fiber sensor integrated with a cylindrical elastomer for in-situ monitoring high-voltage cable accessories for safety work.

## 1. Introduction

In a high-voltage power transmission system, the cable joints and terminals are the most important cable accessories because they largely determine the system’s operational reliability. Therefore, it is necessary to in-situ monitor the insulating health of the cable accessories for the safety work of the entire high-voltage cable system [1]. However, because of the imperfect man-made installation of cable accessories in the insulating layer, the cable joints or cable terminals may produce partial discharge (PD) preceding the breakdown [2,3]. As the performance of insulating layer degrades, the intensity of PD will increase quickly; compared with the early stage PD, the destruction of PD will be more significant. The high-voltage cable accessories will slowly break down due to PD and eventually lead to paralysis of the power supply system [4,5]. Therefore, the accurate and reliable detection of PD can determine the power system's operating condition by the use of in-situ monitoring.

Currently, the methods of PD detection are mainly divided into two: electrical and non-electrical measurement [6,7,8]. Many electrical measurement methods are widely used, such as the electromagnetic wave coupling method, the capacitance coupling method, and the Ultra High Frequency (UHF) method. However, electrical methods require a complicated installation of ground wire on detection devices. The acoustic emission method is representatively and widely used as non-electrical measurement. Compared with electrical measurement, acoustic emission measurement is ideal, as they are with little influence on the detected cable equipment and immune to electromagnetic interference.

As is known, the traditional technique of the detection of acoustic emission generated from partial discharge is a piezoelectric transducer (PZT) that has a high sensitivity, but the huge electromagnetic stresses often limit it from being used widely [9]. Therefore, acoustic sensors based on optical fiber interference are presented, which have the property of immunity from electromagnetic interferences [10]. Recently, a number of fiber optic sensors with various structures have been developed to detect acoustic emission signals from PD, including Faber-Perot interferometer sensors [11,12,13], fiber Bragg grating (FBG) sensors [14,15], Sagnac interferometer sensors [16,17], and the interference sensor based on fiber lasers [18,19].

In this paper, we demonstrate a fiber-optic Michelson interferometric acoustic sensor based on a cylindrical elastomer with a diameter of 3 cm and a thickness of 2 cm. The acoustic emission pressure is applied to the sensing fiber through the elastomer, resulting in the output intensity changing of the interferometer. The sensor head exhibits advantages including a compact size, a relatively high sensitivity, and a broadband frequency response. The sensitivity of the sensor is more than 1.7 rad/(m⋅Pa) at 20 kHz, and a signal-to-noise ratio (SNR) of 66.7 dB is achieved at 20 kHz. The SNR of the sensor head is at a flat response ranging from 20 kHz to 150 kHz. The demonstrated PD sensor technology presents a relatively high sensitivity. Moreover, it can easily realize remote and multi-point sensing network, and the sensor head does not need a power supply and ground wire. These advantages entail the feasibility of the application of safety monitoring in high-voltage cable accessories.

## 2. Sensing Principle

**Figure 1:**
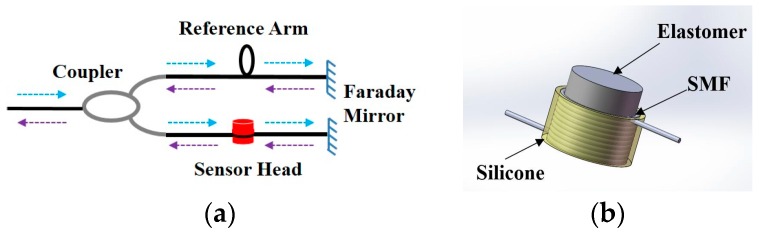
Schematic diagram of (**a**) the Michelson interferometer-based fiber sensor and (**b**) the sensor head constructed by winding optical fiber on the elastic cylinder: the single-mode fiber (SMF).

The purpose of the fiber sensor is to detect the acoustic emission generated by the partial discharge in high-voltage cable accessories. When the acoustic emission signal acts on the sensing head, it transmits in the form of sound pressure that can change the optical path length of the sensing fiber, and thus the phase of light propagating in the fiber. Therefore, the working principle of the sensor is to demodulate the phase vibration of light in the sensing fiber in order to retrieve the acoustic emission signal. Since the probe-type fiber-based interferometer sensors can transmit and collect the signal in the same fiber, it can achieve long-range detection. Here, we utilized a Michelson interferometer-based fiber sensor to detect the acoustic emission, which is schematically shown in Figure 1a. The sensor consists of a coupler and two fiber arms with two mirrors, respectively. The effect of the dependent polarization phenomenon is considered, which might cause the polarization induced fading and the input polarization-induced phase noise. We use the Faraday rotator mirrors (FRMs) in two arms of the Michelson interferometer rather than the common mirrors without polarization control. For FRMs, with a signal pass rotation of 90°, the polarization evolution in the fiber in one direction is essentially “unwound” in the reverse direction, producing a returned state of polarization that is stable in time irrespective of the birefringence properties of the fiber link [20]. The optical beam from the light source is split into two paths by the optical coupler. It exists between the arms of modulation with the measured phase difference, wherein the arm of the reference arm, the other arm, is the sensing arm affected by the acoustic emission signal. Two beams of light reflect back through the FRMs and the interference occurs through the coupler.

Since the acoustic pressure waves change the optical path length of the sensing fiber in the interferometer, the phase variation of the system is coded with the information of the acoustic pressure waves. Through the optical fiber length L, the phase of the sensing fiber (ϕ) is given by:

(1) $$\phi =\beta L\left(t\right)=\frac{2{\pi n}_{\mathrm{eff}}}{\lambda }L\left(t\right)$$

where β is the propagation constant, $n_{\mathrm{eff}}$ is the effective refractive index of the fiber, and λ is the optical wavelength. Since the optical fiber being disturbed by acoustic emission pressure, the amount of change in the phase is as follows:

(2) $$\Delta \phi =\beta \Delta L\left(t\right)+\Delta \beta L\left(t\right)=\Delta \phi_{1}+\Delta \phi_{2}$$

in which the phase ($\Delta \phi_{1}$) refers to the axial stretching of the fiber and is given by:

(3) $$\Delta \phi_{1}=-\frac{\beta L\left(t\right)}{E}\left(1-2\upsilon \right)\Delta P$$

where υ is the Poisson ratio, E is the Young modulus, and ΔP is the acoustic pressure change. The phase ($\Delta \phi_{2}$) in Equation (2) is the change of the propagation constant that depends on the change of the effective refractive index (n) due to the elastic-optic effect and the fiber diameter produced by the strain. However, the effect of the change on the diameter is proved to be negligible [21], so ($\Delta \phi_{2}$) can be written as:

(4) $$\Delta \phi_{2}=-\frac{\beta L\left(t\right)}{2E}\left(1-2\upsilon \right)\left(p_{11}+2p_{12}\right)\Delta P,$$

where $p_{11}$ and $p_{12}$ are elements of the strain-optic tensor.

As given in Equation (3), in order to achieve high sensitivity to acoustic pressure change, a large Poisson ratio (υ) is preferred. In the work, we propose a simple sensor head with a high Poisson ratio. A piece of fiber is wound around the elastic cylinder carefully and softly as shown in Figure 1b. The silicon rubber glue is coated on the wound fiber to fix the fiber firmly. Such a sensor head has advantages of little negative influence on the electrical properties of the high-voltage cables and can withstand a high external pressure strength. The standard single-mode fiber (SMF) is chosen as the sensing fiber because of its compatibility with the traditional fiber link and capability of mass production. In order to improve the sensitivity of the system, the material of elastic cylinder is selected to be silicone rubber with a high Poisson's ratio of 0.49 [22,23]. In addition to the material of the elastic cylinder, the sensitivity of the sensor is determined by the geometric transformation of the elastic cylinder. The bottom of the cylinder is subjected to acoustic pressure waves, and its lateral transformation causes a change in the length of the SMF. On the one hand, the energy of the sound pressure spreads along the elastic cylinder where energy will slowly decay to the directional propagation of sound pressure. Therefore, the optical fiber is wound around the side surface of the elastic body close to the sound source. A height of 2 cm for the elastomer is chosen to ensure the compact design as well as the relatively uniformity of geometric transformation. On the other hand, the fiber bending loss is an important point in choosing the appropriate radius of the cylinder. If the radius of the cylinder is too small, it will increase the transmission loss in the fiber and affect the interference light detection. According to the parameters of the standard single-mode fiber, whose characteristics are defined by ITU-T G.652 (Single-Mode Fiber-SMF) [24], the bending loss of the fiber is less than 0.005 dB per loop [25] at the bending radius of 1.5 mm. Taking the fiber bending loss into account, the 1.5 cm radius of the cylindrical elastomer is used in the production of the sensor head. With an increasing number of turns wrapped around the elastomer, the sensitivity of the sensor head will improve. The elastomer is wrapped 15 times, i.e., the length of the fiber is approximately 1.5 m, which guarantees a small compact sensor head. During the experimental investigation, despite a high Poisson’s ratio, the geometry deformation of the elastic cylinder at different acoustic pressure levels is actually tiny, which leads to a weak force to expand the length of the wrapped fiber. Therefore, the reversed force by the fiber loops is not strong enough to permanently deform the polymer. 

## 3. Characterization of Michelson Interferometer-Based Fiber Sensor for Acoustic Emission Detection

### 3.1. Experimental Setup of Michelson Interferometer-Based Fiber Sensing System

**Figure 2:**
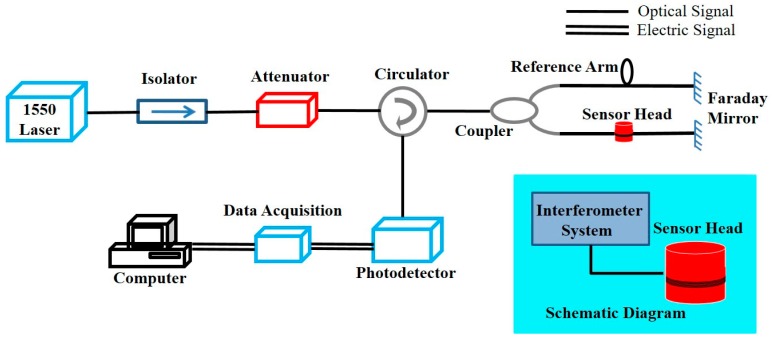
Experimental setup of the Michelson interferometer-based fiber sensing system, the inset shows its equivalent diagram.

The demodulation system of Michelson interferometer-based fiber sensor is shown in Figure 2. A narrow linewidth laser working at a center wavelength of 1550 nm is provided as the light source, which is followed by an isolator to prevent damage to the light source by the optical reflection. An optical circulator collects the reflected light from the sensor head for data analysis. The optical fiber used in the system is a SMF operating at a wavelength of 1550 nm. The photodetector is used to monitor the interference signal by converting the optical signals into electrical signals. After the collecting electrical signal is sampled by a data acquisition card and converted into a digital signal by computer processing, the outcome of data analysis is displayed on the computer. The output light intensity can be expressed as:

(5) $$W_{\mathrm{out}}=\frac{1}{2}W_{0}\left[1+\cos\left(\Delta \varphi \left(t\right)+\varphi_{0}\left(t\right)\right)\right],$$

where Δφ(t) is the optical phase change due to the acoustic wave generated by the partial discharge, $\varphi_{0}\left(t\right)$ is the initial phase when changes are produced, $W_{\mathrm{out}\;}$ is the output intensity of the detector, and $W_{0}/2$ refers to the mean value of the intensity at the detector. 

Due to the surrounding environmental disturbance of the system, the initial phase will be a low-frequency drift. However, the detection of the partial discharge is a high-frequency signal covering the frequency range of 20 kHz to 150 kHz. A high-pass filter and a high-frequency signal amplification were added to data acquisition to reduce the impact of low-frequency drift, which improved the performance of the high-frequency signal detected.

### 3.2. Characterziation of Frequency Response and Sensitivity of the Proposed Sensing System

**Figure 3:**
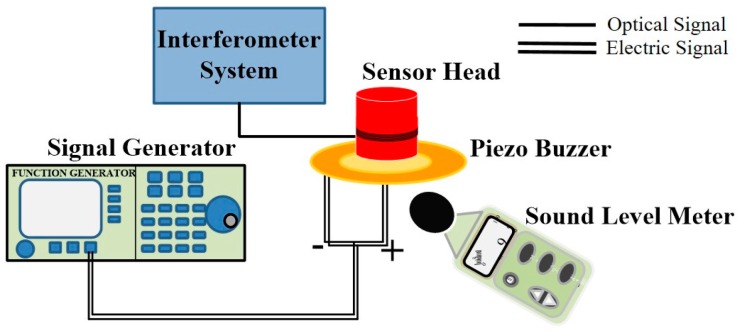
Experimental set-up for the calibration of the sensor.

For the Michelson interferometer-based phase fiber sensor, the phase of light propagating along the fiber is directly modulated by the acoustic pressure. Therefore, it is preferable to detect the phase change of light to calibrate its sensitivity [4]. Based on the demodulation system of the Michelson interferometer-based fiber sensor shown in Figure 2, the characterization of frequency response and sensitivity is conducted in the laboratory environment as shown in Figure 3. The related equipment contains an interferometer system with a sensor head and a handheld sound level meter (SLM), which is commonly used in acoustic measurement. A piezo buzzer excited by a signal generator is used as the acoustic source, which can generate sinusoidal acoustic waves in a specific frequency. The SLM is used to calibrate the sound pressure.

**Figure 4:**
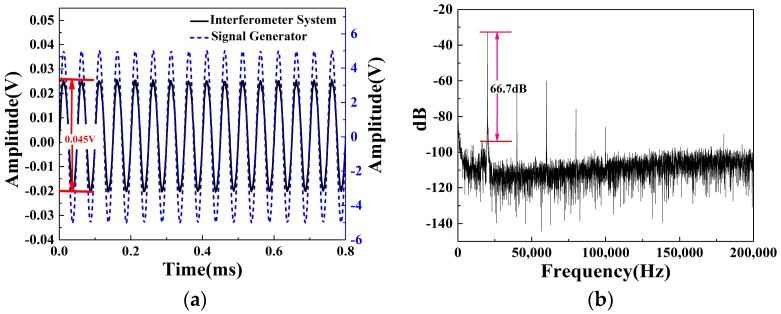
(**a**) The same acoustic emission detected with the interferometer system and the signal generator; (**b**) The detected acoustic frequency of the interferometer system.

Figure 4a,b show the response results of the proposed sensor in both time and frequency domain at 20 kHz under an acoustic pressure level of 79 dB. The acoustic pressure is generated by the piezo buzzer measured by the SLM. Since the sound pressure level ($L_{P}$) is measured in a logarithmic scale, we can deduce $L_{P}$ in a linear scale as follows [26]:

(6) $$L_{P}\left(\mathrm{dB}\right)=20\mathrm{lg}\left(\frac{P}{P_{0}}\right)$$

where P is the sound pressure, and $P_{0}$ is the reference sound pressure where $P_{0}=2\times 10^{-5}\;\mathrm{Pa}\;$in the air. Therefore, the sound pressure on the sensor head is estimated to be 0.18 Pa$\;\left(=10^{79/20}\times 2\times 10^{-5}\;\mathrm{Pa}\right)$.

According to Equation (5), the optical phase change originating from the vibration of acoustic pressure can induce the optical intensity change in the interferometer sensor. In order to manifest the optical sensitivity of the proposed sensor, we can retrieve the optical phase change information from the optical intensity interference that is converted into the electrical voltage measured by the photodetector as shown in Figure 2. By manipulating the acoustic pressure level, the output peak-to-peak value of the electrical signal is maximized to be 0.1 V, corresponding to the phase change of π. Figure 4a shows when the signal generator generates the sine wave with an amplitude of 5 V, the output signal of the interferometer system follows the same trace with a peak-to-peak value of 0.045 V. Therefore, the output peak-to-peak value of 0.045 V driven by the applied AC voltage of 5 V corresponds to a phase change of ±0.47 rad $\left(=\pm \sin^{-1}\left(0.045\;V/0.1\;V\right)\right)$. The phase shift per unit length and unit pressure of this sensor is calculated to be 1.7 rad/(m⋅Pa) (=0.47 rad/1.5 m/0.18 Pa) at a 20 kHz measurement frequency, which can be regarded as the sensitivity of the sensor [27]. Therefore, the fiber optic sensor head has a sensitivity of 1.7 rad/(m⋅Pa), which is relatively higher than the sensitivity of the sensors shown in a previous report of 40 μrad/(m⋅Pa) [4]. If the sensitivity is calculated in terms of mV, because the peak-peak value of 0.045 V corresponds to a phase change of 0.47 rad, we can convert the value of 1.7 rad/(m⋅Pa) to 163 mV/(m⋅Pa). A signal-to-noise ratio (SNR) of 66.7 dB with a noise background of about −100 dB can be observed in Figure 4b.

**Figure 5:**
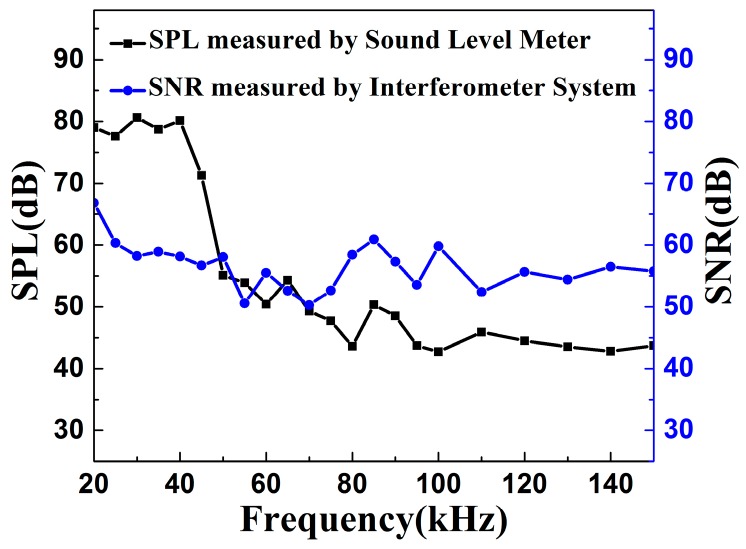
The relationship between frequency and the signal-to-noise ratio (SNR) of the sensor system.

The frequency response of the interferometer system is further investigated by driving the piezo buzzer with frequency sweeping in the range of 20–150 kHz. The relationship between frequency and the SNR of the sensor system is shown in Figure 5. The acoustic pressure level of 79 dB is observed at 20 kHz measured by the sound level meter. Because of the intrinsic frequency response of the piezo buzzer is better in low frequency than that in high frequency, the sound pressure level of the acoustic signal generated by the piezo buzzer has a significant decline with increasing frequency. Different from the sound pressure level of the sound source, which has a significant decline, the SNR of the sensor head is at a flat response ranging from 20 kHz to 150 kHz. Especially, the high SNR of the sensor head is maintained at a strong sound pressure because the mechanical deformation of the elastomer increases the fiber length changes. Compared with fiber sensors shown in previous reports for PD detection [21], our developed fiber sensor system shows a higher SNR and a wider frequency response. Such a frequency window covers the frequency components of acoustic emission generated by a partial discharge, which is the base of in-situ monitoring for safety work of the accessories of a high-voltage cable.

## 4. Detection of Acoustic Emission by High Voltage Partial Discharge

### 4.1. Dectection of Partial Discharge in the Laboratory Environment

**Figure 6:**
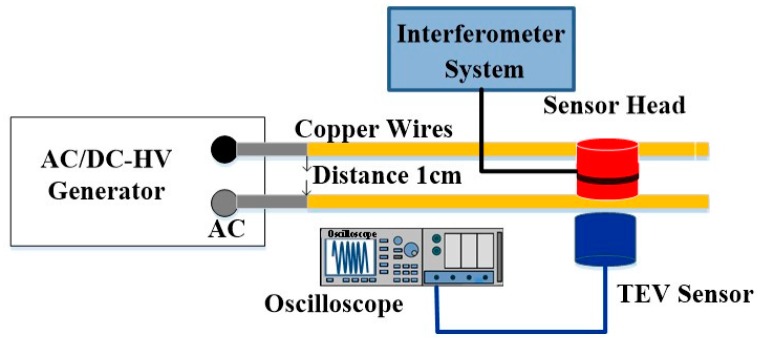
Experimental setup of the partial discharge of copper wires.

We firstly detected PD acoustic signals through AC high voltage with two parallel copper wires in the laboratory. This experimental work was to verify the feasibility of the optical sensor head and the sensitivity of the developed sensor. The experimental setup for PD detection is shown in Figure 6, in which the optical sensor head is placed on the copper wires. The devices for the optical measurement part are given in Figure 2. Such PD signals were also detected by the utilization of an electrical sensor based on the transient earth voltage (TEV) method. The TEV sensor is a commercial product made by the Hanbit EDS Company (Daejeon, Korea). It is a kind of resonance capacitive coupling sensor with a bandwidth of 1–5 MHz. The distance of the copper wires was fixed at 1 cm, and the diameter of the used copper wires was 2 mm. The imposed AC high voltage produced by the AC/DC-HV generator was adjustable from 0 V to 8 kV. In this experiment, when the peak-to-peak voltage reaches about 4 kV, the partial discharge occurred periodically. 

**Figure 7:**
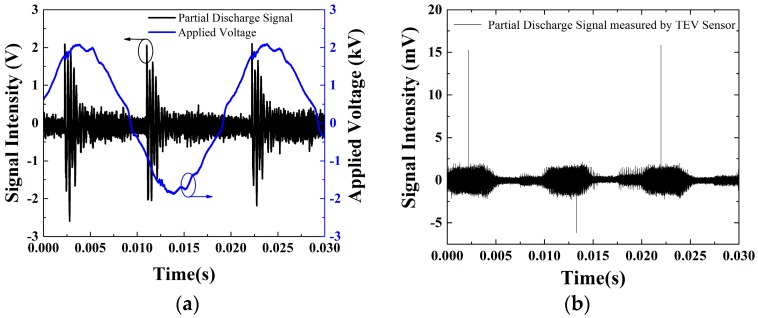
(**a**) The applied voltage and the partial discharge signal detected by the interferometer system; (**b**) The partial discharge signal detected by the transient earth voltage (TEV) sensor.

The applied signal as well as the PD signal measured by the developed optical sensor based on the interferometer system is shown in Figure 7a. The 2 kV of applied signal only represents the peak voltage. The voltage of the PD signal measured by the optical sensor system shows a peak-to-peak value of 4 V. It should be noted that the occurrence of the partial discharge is directly related to the phase of the alternating voltage imposed on the copper wires. Signals were detected at intervals of approximately 10 ms, which corresponds to a half cycle of AC voltage with a frequency of 50 Hz. It is well known that PDs usually ignite on the rising positive half cycle, and the decreasing negative half cycle for the high-voltage AC cables generates a partial discharge [22,28,29]. The reason is that the points of partial discharge will have a polar effect at high-voltage AC. When the electric field just exceeds the critical field strength, a strong discharge occurs and the space charge around the discharge point increases with time. Therefore, the discharge pulse concentrates in the rising positive half cycle and the decreasing negative half cycle.

In order to assess the level of PD generated from the copper wire source, we used the TEV sensor, and the measurement results are given in Figure 7b. It is observed that impulse signals occur at intervals of approximately 10 ms. This observation is consistent with the signals that are measured by the optical sensor as shown in Figure 7a. In Figure 7b, the peak-to-peak value of the PD signal detected by the TEV sensor is up to ~15.8 mV. Generally, the measured value of TEV will be displayed in dB as follows [30,31]:

(7) $$\mathrm{dB}=20\mathrm{lg}\left(\frac{U}{U_{0}}\right)$$

in which U refers to the amplitude measured by TEV sensor, and $U_{0}$ represents pedestal voltage. For the applied TEV sensor, the pedestal voltage is about 1 mV. Therefore, 15.8 mV corresponds to ~24 dB. For the same PD source, the peak-to-peak value of the PD signal detected by our optical sensor is ~4.5 V as shown in Figure 7a. The pedestal voltage level is ~400 mV as shown in Figure 7a. Therefore, 4.5 V corresponds to 21 dB according to Equation (7). This indicates that the performance of the developed optical sensor is comparable to that of the commercial TEV sensor for the detection of PD signals. The main advantage of optical fiber sensors over the traditional electrical method is anti-electromagnetic interference.

### 4.2. Detection of Real Partial Discharge Generated in Gas Insulated Switchgear (GIS)

**Figure 8:**
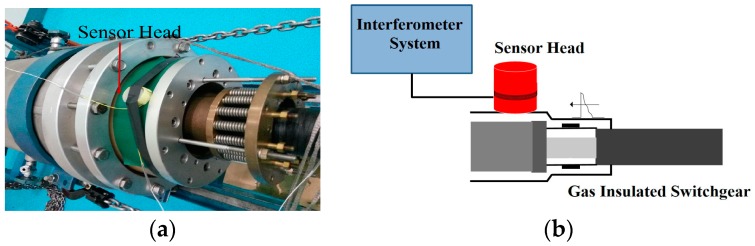
Photograph and experimental setup of the 220 kV model GIS, (**a**) a photograph of 220 kV GIS; (**b**) Experimental setup of the 220 kV model Gas Insulated Switchgear (GIS).

The sensor head was manufactured and tested with real partial discharge generated in Gas Insulated Switchgear (GIS). The partial discharge was generated by putting a small metal chip in the GIS. This experimental work was to verify the feasibility of the sensor head and the sensitivity of the acoustic emission of partial discharge in real applications. Figure 8a shows a photo of the GIS that is imposed AC high voltage, and Figure 8b is the schematic diagram. The sensor head was attached on the GIS, and the sensor head was about 10 cm from the source of the partial discharge. The AC high voltage was generated by an HV transformer with a variable AC low-voltage input.

**Figure 9:**
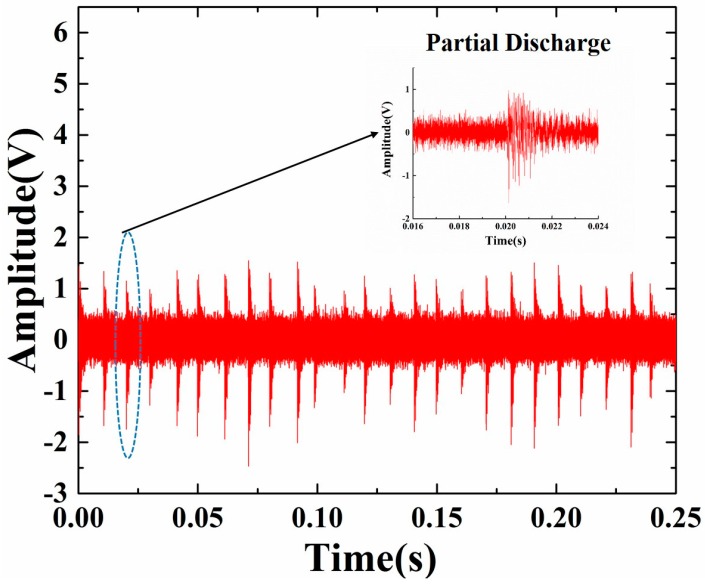
Experimental results on the sensing of partial discharge in GIS.

The detected signals that occurred when the AC high voltage imposed on the electrodes reached about 80 kV are shown in Figure 9. The figure shows that the detected signal interval was 10 ms, which proves that the signal resulted from partial discharge. Because of the addition of metal shavings in the GIS, field stress is not uniform and discharge occurs locally. At each time, a partial discharge occurs, and the state and size of the metal shavings will change slightly. As a result, the detected signal strength is floating. The sensor head shows enough sensitivity to detect the acoustic emission of the partial discharge. The experimental results presented in Figure 9 illustrate the feasibility and practicability of the sensor head with a detection distance of 10 cm, which might have a potential application in power systems for the PD monitoring of high-voltage cable accessories.

Our sensor head has potential applications in the terminals of high-voltage cables whose physical length is relatively short. In order to overcome the limitation that the partial discharge signal attenuates in space and extends the detection capacity in distance, we can build the sensor network with more than two sensor heads to achieve the distributed acoustic pressure sensing.

In real applications, a change in environmental temperature may induce the deformation of the polymer due to the thermal-expansion effect. Such a deformation of the polymer might induce frequency drift of the entire interferometer-based fiber sensor. However, the change time of the environmental temperature is generally far slower than the change time of the detected acoustic pressure. Therefore, we can use a filter in the detection to suppress the cross-sensitivity induced by the temperature. If the temperature change is extremely intense and fast (less than ms), we can compensate the temperature change-induced cross sensitivity by wrapping the fiber on another identical polymer in the reference arm of the Michelson interferometer. The key is to isolate this reference arm integrated with the polymer from the acoustic emission source. 

## 5. Conclusions

In this work, we propose and demonstrate a Michelson interferometry-based fiber sensor for detecting acoustic emissions generated from the partial discharge. The developed sensor was integrated with a cylindrical elastomer, showing a relatively high sensitivity and broadband frequency response. The sensitivity of our developed sensor for acoustic detection of partial discharges is calculated to be 1.7 rad/(m⋅Pa). Moreover, a high frequency response up to 150 kHz is achieved. The relatively high sensitivity for the detection of PD was verified in both the laboratory and with GIS. Moreover, an electrical sensor-based TEV method was utilized to assess the level of PD from the source. The results show that the performance of the developed optical sensor is comparable to that of the commercial TEV sensor for the detection of PD signals. Since the fiber sensor head has a compact design and is capable of mass production, it has the potential to meet the needs of remote and on-line partial discharge detection for the safety work at the GIS. Different from electrical PD measurement, for example, the coupling capacitor method, which has poor anti-jamming performance, is susceptible to external electromagnetic interference, and is difficult to achieve on-line monitoring, our developed sensor head behaves as a sound-to-electrical converter that is immune to electromagnetic interference, capable of remote monitoring, and capable of constructing sensor networks.
